# Effects of Venoconstrictive Thigh Cuffs on Dry Immersion-Induced Ophthalmological Changes

**DOI:** 10.3389/fphys.2021.692361

**Published:** 2021-07-14

**Authors:** Marc Kermorgant, Ayria Sadegh, Thomas Geeraerts, Fanny Varenne, Jérémy Liberto, François-Philippe Roubelat, Noémie Bataille, Marie-Pierre Bareille, Arnaud Beck, Brigitte Godard, Adrianos Golemis, Nathalie Nasr, Dina N. Arvanitis, Ophélie Hélissen, Jean-Michel Senard, Anne Pavy-Le Traon, Vincent Soler

**Affiliations:** ^1^INSERM DR Midi-Pyrénées Limousin, Institute of Cardiovascular and Metabolic Diseases (I2MC) UMR1297, University Hospital of Toulouse, Toulouse, France; ^2^Department of Ophthalmology, University Hospital of Toulouse, Toulouse, France; ^3^Department of Anaesthesiology and Critical Care, University Hospital of Toulouse, Toulouse, France; ^4^Institute for Space Medicine and Physiology (MEDES), Toulouse, France; ^5^Department of Neurology, University Hospital of Toulouse, Toulouse, France; ^6^Department of Clinical Pharmacology, University Hospital of Toulouse, Toulouse, France

**Keywords:** optical coherence tomography, optic nerve sheath diameter, intraocular pressure, intracranial pressure, dry immersion, microgravity, countermeasures, thigh cuff

## Abstract

Neuro-ophthalmological changes named spaceflight associated neuro-ocular syndrome (SANS) reported after spaceflights are important medical issues. Dry immersion (DI), an analog to microgravity, rapidly induces a centralization of body fluids, immobilization, and hypokinesia similar to that observed during spaceflight. The main objectives of the present study were 2-fold: (1) to assess the neuro-ophthalmological impact during 5 days of DI and (2) to determine the effects of venoconstrictive thigh cuffs (VTC), used as a countermeasure to limit headward fluid shift, on DI-induced ophthalmological adaptations. Eighteen healthy male subjects underwent 5 days of DI with or without VTC countermeasures. The subjects were randomly assigned into two groups of 9: a control and cuffs group. Retinal and optic nerve thickness were assessed with spectral-domain optical coherence tomography (OCT). Optic nerve sheath diameter (ONSD) was measured by ocular ultrasonography and used to assess indirect changes in intracranial pressure (ICP). Intraocular pressure (IOP) was assessed by applanation tonometry. A higher thickness of the retinal nerve fiber layer (RNFL) in the temporal quadrant was observed after DI. ONSD increased significantly during DI and remained higher during the recovery phase. IOP did not significantly change during and after DI. VTC tended to limit the ONSD enlargement but not the higher thickness of an RNFL induced by DI. These findings suggest that 5 days of DI induced significant ophthalmological changes. VTC were found to dampen the ONSD enlargement induced by DI.

## Introduction

During the last decade, some studies reported ophthalmic abnormalities in astronauts who spent several months onboard the International Space Station (Lee et al., 2016). Today, these neuro-ophthalmological changes have been more clearly defined as spaceflight associated neuro-ocular syndrome (SANS). After long-duration flights, some astronauts exhibited persistent ophthalmologic disorders characterized by hyperopic shift. High inter-individual variability in ophthalmological findings was observed, such as an enlargement in optic nerve sheath diameter (ONSD), papilledema, globe flattening, increase in circumpapillary retinal nerve fiber layer thickness (RNFLT), and optic disc edema (Mader et al., 2011; Kramer et al., 2012; Laurie et al., 2020; Lee et al., 2020; Macias et al., 2020). Recent studies using optical coherence tomography (OCT) have shown some level of optic disc edema in nearly all astronauts when comparing pre-flight and in-flight OCT, suggesting that subclinical SANS involvement may occur in the majority of astronauts (Lee et al., 2020). Some of these changes are similar to those observed in idiopathic intracranial hypertension (Nelson et al., 2014). However, the pathophysiology of SANS remains unresolved. One previous hypothesis suggested localized events occurring at the level of the intra-orbital optic nerve with the possible implication of elevated intracranial pressure (ICP) (Roberts et al., 2017; Zhang and Hargens, 2018; Mader et al., 2019).

Analogs of microgravity such as head-down bed rest (HDBR) and dry immersion (DI) are valuable models for determining the effects of spaceflight on the health of the astronauts; however, in contrast to the other ground-based models of microgravity, DI is renowned for its effectiveness by eliciting rapid physiological changes (Tomilovskaya et al., 2019). In a short period, DI mimics the absence of any supporting structure for the body, immobilization, hypokinesia, and centralization of body fluids by transmural hydrostatic pressure, observed during human spaceflight (Navasiolava et al., 2011). DI impacts a wide range of physiological mechanisms in particular a potential impact on ICP (Arbeille et al., 2017; Kermorgant et al., 2017).

Several preventive countermeasures (nutritional supplementation, muscular exercise, thigh cuffs, etc.) have been tried to prevent post-flight orthostatic intolerance, yet most of these have shown only partial efficacy. In the 1960s, several studies focused on specific venous occlusion bracelets named venoconstrictive thigh cuffs (VTC) with a beneficial decrease of facial edema and congestion (Lindgren et al., 1998). VTC are strips worn around thighs for several hours per day. These countermeasures are commonly used by cosmonauts to mitigate the symptoms related to the headward fluid shift. VTC have also been applied during ground-based models of microgravity whereby they were shown to dampen hypovolemia after microgravity exposure but failed to prevent orthostatic intolerance (Arbeille et al., 1999; Custaud et al., 2000; Millet et al., 2000; Pavy-Le Traon et al., 2001).

This study aimed to evaluate the neuro-ophthalmological changes induced by a 5 day DI and to determine the potential beneficial effects of VTC on these DI-induced ophthalmological adaptations. We postulated that DI-elicited cephalad fluid shift will result in ICP increase as reflected by ophthalmological changes and that VTC countermeasures could dampen the effects of DI on ICP by mitigating the cranial fluid shift.

## Materials and Methods

### Subjects

The clinical trial (ID-RCB 2018-A01470-55; Clinical Trial Identifier: NCT03915457) was conducted in accordance with the principles laid down by the Declaration of Helsinki after approval by both the CPP Est III Ethics Committee (October 2, 2018) and the French Health Authority, ANSM (August 13, 2018). Twenty healthy men participated in the study and gave their written consent. Two subjects withdrew before the 5 days of ambulatory baseline data collection (BDC) for reasons unrelated to the protocol. At BDC-2, 18 subjects were included in the study and randomly allocated into two groups of 9: a control (34 ± 7 years; 176 ± 6 cm; 74 ± 8 kg) and cuffs (34 ± 4 years; 180 ± 4 cm; 74 ± 9 kg) group. The inclusion and non-inclusion criteria are presented in Table 1.

**Table 1:** Inclusion and non-inclusion criteria.

**Inclusion criteria**	**Non-inclusion criteria**
• Healthy male participant, age between 20 and 45 years, height between 158 and 185 cm, body mass index (BMI) between 20 and 26 kg/m^2^. • No personal nor family record of a chronic or acute disease or psychological disturbances. • Fitness level assessment: ✓ if age < 35 years: 35 ml/min/kg < VO_2_ max < 60 ml/min/kg. ✓ if age > 35 years: 30 ml/min/kg < VO_2_ max < 60 ml/min/kg. • Active and free from any orthopedic, musculoskeletal, and cardiovascular disorders. • No history of regular smoking, no alcohol, no drug dependence, and no medical treatment (2 months before the beginning of the study).	• Past record of orthostatic intolerance, arterial hypertension, and cardiac rhythm disorders. • Chronic back pains, vertebral fracture, scoliosis or herniated disc, history of knee problems, or joint surgery/broken leg. • History of hiatus hernia or gastro-esophageal reflux, thyroid dysfunction, renal stones, diabetes, glaucoma, and migraines. • Past records of thrombophlebitis, family history of thrombosis, or positive response in the thrombosis screening procedure. • Abnormal result for lower limbs in Doppler ultrasound. • Bone mineral density: T-score ≤ −1.5, osteosynthesis material, presence of metallic implants. • Poor tolerance to blood sampling and having donated blood (more than 8 ml/kg) in a period of 8 weeks or less before the start of the experiment.

### General Protocol

**Figure 1:**
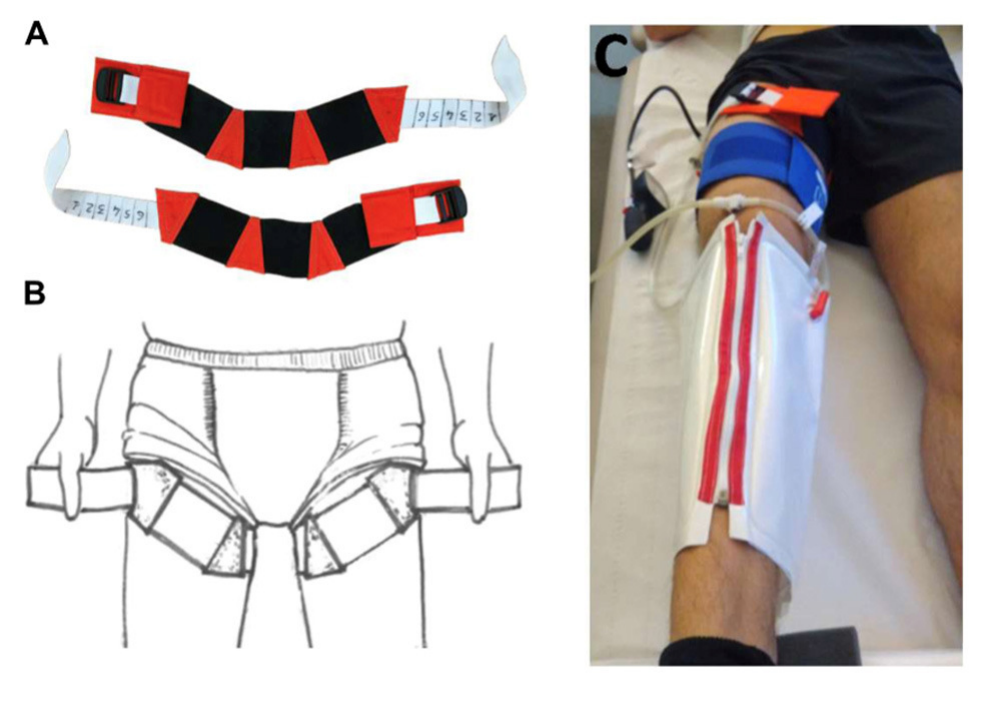
Venoconstrictive thigh cuffs (VTC) countermeasure (photo MEDES). **(A)** Venoconstrictive thigh cuffs are elastic strips, adjustable to the size of the thigh with clamping segment (white segment). **(B)** VTC are worn on the upper part of the thigh. **(C)** Individual adjustment of VTC with plethysmography to apply a 30 mmHg pressure on the upper thigh and performed at BDC-2.

**Figure 2:**
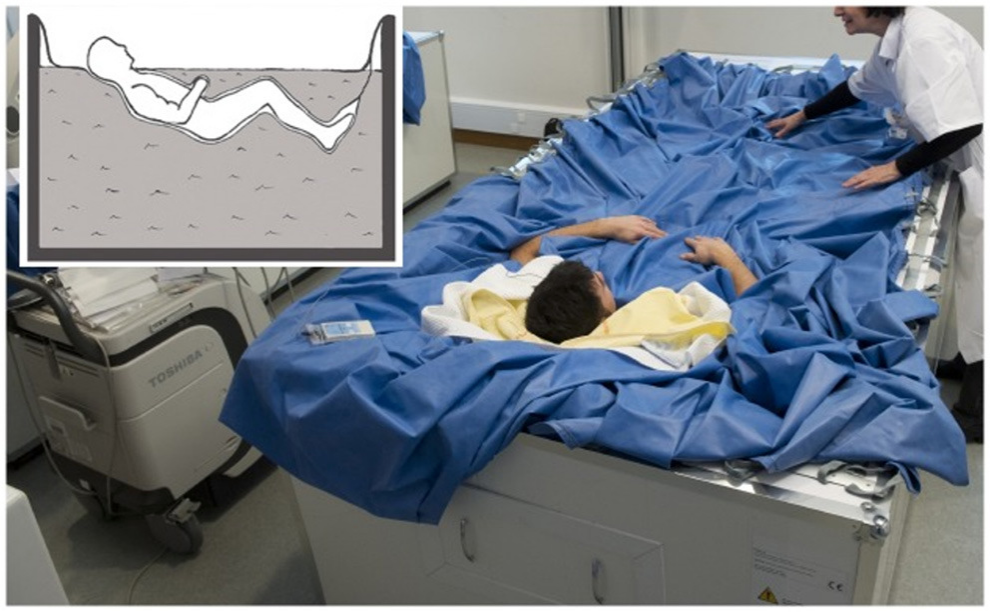
The subject was immersed slowly in a supine position covered with an elastic waterproof fabric in MEDES dry immersion (DI) facility.

**Figure 3:**
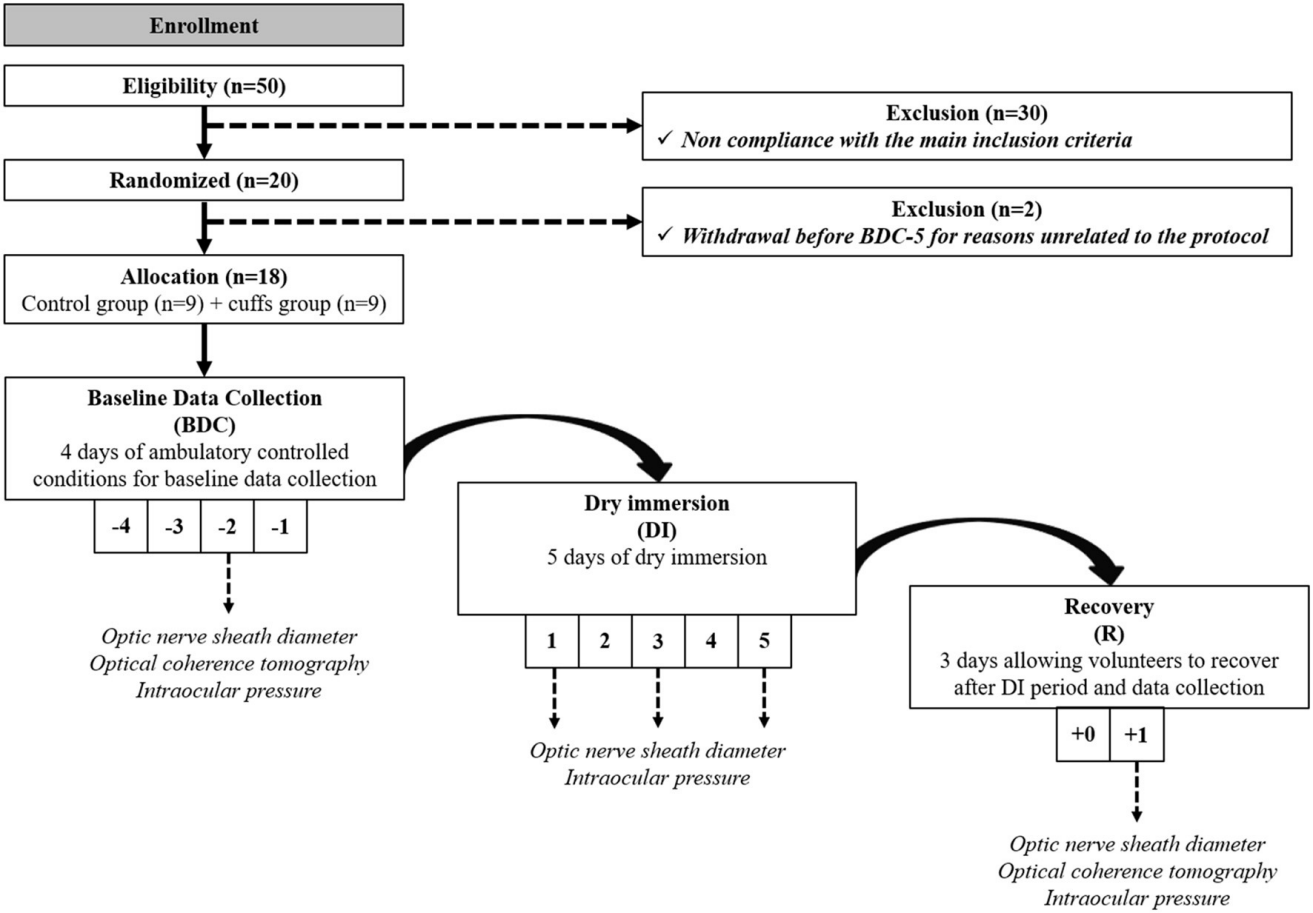
Flow chart of the study and timeline of ophthalmological data collection.

The study was carried out at the Institut de Médecine et de Physiologie Spatiales (MEDES) in Toulouse, France, between November 2018 and March 2019. The detailed protocol was described by Robin et al. (2020). The protocol included 4 days of ambulatory BDC before DI (BDC-4 to BDC-1), 5 days of DI (DI 1 to DI 5), and 2 days of ambulatory recovery (R 0 to R+1). Subjects included in the cuffs group wore VTC throughout DI (from 10:00 a.m. to 06:00 p.m. at DI 1 and from 08:00 a.m. to 06:00 p.m. at DI 2–DI 5). At DI 1 (10:00 a.m.), VTC were placed on subjects immediately prior to immersion. VTC was adjusted for each subject to obtain an occlusion pressure of about 30 mmHg (Figure 1). Briefly, the DI model consists of placing the body of the subject in thermoneutral water (32–34.5°C) covered with an elastic waterproof fabric; thus, the subject is in a semi-recumbent position and freely suspended while remaining dry (Figure 2). The study was carried out in a quiet room and where the air temperature was ~24°C. All subjects remained under continual medical observation. The subjects woke up at 07:00 a.m., and the light was switched off at 11:00 p.m. The subjects were allowed to get out of the DI container for daily hygiene procedures, weighing, and other specific measurements. During this period, subjects were maintained in a six degree HDBR position. Overall, out-of-bath time for the 120 h of immersion was 9.7 ± 1.3 h. Each subject had a daily medical follow-up including body weight, blood pressure (BP), heart rate (HR), and tympanic body temperature by permanent MEDES staff. A flow chart of the study is presented in Figure 3.

### Hemodynamics Parameters

Continuous finger BP (Nexfin, BMeye, US) and standard ECG (Biopac, ECG 100C, US) were non-invasively monitored and recorded.

### Optical Coherence Tomography

**Figure 4:**
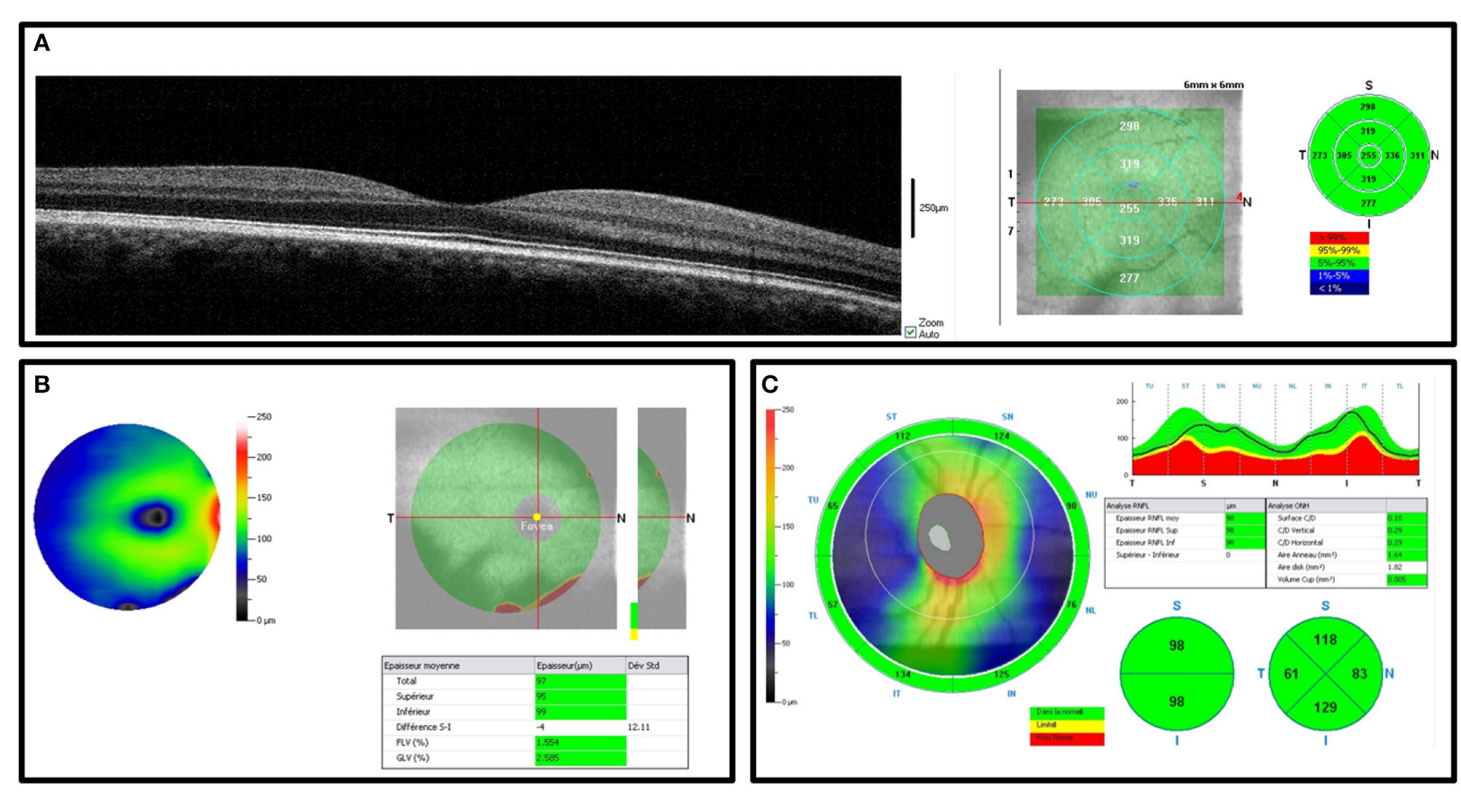
Spectral-domain OCT showing the right eye in one subject before DI. **(A)** Macular thickness. **(B)** Ganglion cell complex (GCC) and significance maps. **(C)** Optic nerve head (ONH) and retinal nerve fiber thickness.

Optical coherence tomography was performed at rest during BDC-2 and R+1 by trained ophthalmologists. OCT was performed with iVue spectral-domain OCT (Optovue iVue®, Fremont, CA). The quality for each measurement was determined by a quality index provided by the OCT device. Measurements not fulfilling this condition were automatically eliminated and repeated. The right and left eyes were assessed. The final measure corresponds to the average of the two measures. All OCT measurements were validated by an expert (Vincent Soler) blinded to the condition. The following parameters were measured:

✓ The retinal map corresponded to the average macular thickness (Figure 4A)
✓ Ganglion cell complex (GCC) thickness was divided into three measurements: average, superior- and inferior-retina (Figure 4B)
✓ Optic nerve head (ONH) thickness was divided into four quadrants: temporal, nasal, superior, and inferior (Figure 4C)

Retinal map and GCC images were acquired on a 6 × 6 mm mapping square centered on the fovea, with 5 to 10 μm resolution horizontal B-scans. ONH and RNFLT measures were acquired with horizontal B-scans centered on the optic disc. Segmentation of the internal limiting membrane and the Bruch membrane opening were verified manually.

### Indirect Assessment of ICP by Ocular Ultrasonography

**Figure 5:**
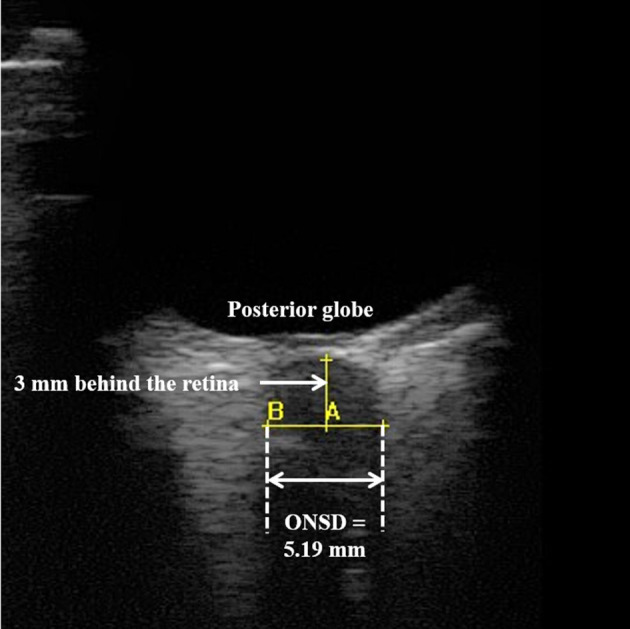
Two-dimensional ocular ultrasonography. Optic nerve sheath diameter (ONSD) was measured 3 mm behind the posterior globe.

Ocular examination was performed at rest during BDC-2, DI 1, DI 3, DI 5, and R+1 by investigators trained for ocular ultrasonography. Ultrasound was performed with a linear high-frequency probe (Orcheo^Lite^, Sonoscanner, Paris, France). First, a thick layer of gel was applied over the closed upper eyelid. The probe was placed on the eyelid and adjusted to obtain an appropriate display of the optic nerve into the globe. The assessment was realized in a two-dimensional mode and ONSD was measured 3 mm behind the ocular globe (Figure 5). The right and left optic nerves were assessed, and one measure was performed for each eye in the sagittal plane. The final measure corresponds to the average of these two measures. All ONSD measurements were validated by an expert (Thomas Geeraerts) blinded to the condition.

### Intraocular Pressure (IOP) by Applanation Tonometry

A topical local anesthetic solution (Tetracaïne, 1% per ophthalmic drops) was instilled in each eye just prior to evaluation. The IOP measurements were performed in both eyes with a Tono-Pen® tonometer during BDC-2, DI 1, DI 3, DI 5, and R+1. The final measure corresponds to the average of these two measures.

### Statistical Evaluation

The primary endpoint was the ONSD increase under DI. Based on the previous study (Kermorgant et al., 2017), we suggested that VTC will limit the ONSD increase. A limitation of ONSD increase by 50% seemed clinically relevant. With a statistical power of (1–β) 90% and α risk of 0.05 in a bilateral hypothesis, we hypothesized that for a 50% change in ONSD, we needed 20 participants (10 in a control group and 10 in the cuffs group). Hemodynamics parameters, OCT, ONSD, and IOP data were expressed as mean ± SD. The normality of the distributions was assessed with the Shapiro-Wilk normality test. Two-way repeated-measures ANOVA was used with Dunnett's and Bonferroni's multiple comparisons test. The day of measurements and countermeasure condition were included, respectively, within-subjects and between-subjects factors. Differences were considered statistically significant when *p*-value was adjusted to ≤ 0.05. All statistical analyses were performed with Prism GraphPad 9.0.0.

## Results

### Hemodynamics Parameters

Hemodynamics parameters are summarized in Table 2. Systolic blood pressure (SBP), diastolic blood pressure (DBP), and heart rate (HR) were not significantly modified throughout the experiment both in the control and cuffs groups.

**Table 2:** General hemodynamic parameters.

	**Control**					**Cuffs**				
	***BDC-2***	***DI 1***	***DI 3***	***DI 5***	***R+1***	***BDC-2***	***DI 1***	***DI 3***	***DI 5***	***R+1***
SBP (mmHg)	115 ± 11	111 ± 10	116 ± 12	117 ± 10	119 ± 9	117 ± 10	113 ± 9	112 ± 4	122 ± 7	119 ± 9
	(107–124)	(104–119)	(107–125)	(109–125)	(112–126)	(109–125)	(106–120)	(109–115)	(117–128)	(112–126)
DBP (mmHg)	68 ± 5	66 ± 6	66 ± 6	69 ± 9	70 ± 8	68 ± 9	67 ± 4	68 ± 7	69 ± 8	71 ± 5
	(64–72)	(62–71)	(62–71)	(61–76)	(64–76)	(61–74)	(64–70)	(62–73)	(63–75)	(67–75)
HR (bpm)	57 ± 6	57 ± 8	51 ± 4	57 ± 8	63 ± 5	58 ± 8	59 ± 7	57 ± 10	60 ± 8	64 ± 9
	(52–61)	(51–64)	(48–55)	(50–63)	(59–67)	(52–65)	(53–64)	(49–65)	(53–66)	(56–71)

*BDC-2, 2 days before DI; DBP, diastolic blood pressure; DI 1, first day of DI; DI 3, third day of DI; DI 5, fifth day of DI; HR, heart rate; R+1, first day after DI; SBP, systolic blood pressure. Values in parentheses represent 95% CI of the mean*.

### Optical Coherence Tomography

In the control group, one subject was removed from ONH analysis due to technical glitches. In the cuffs group, one subject was removed from OCT data analysis, and 2 other subjects were also removed from ONH analysis due to low-quality index. Table 3 summarizes the ocular characteristics of the volunteers.

**Table 3:** Optical coherence tomography data.

	**Control**		**Cuffs**		**ANOVA table** ** (condition, time, condition by time)**
	***BDC-2***	***R+1***	***BDC-2***	***R+1***	
**AMT (μm)**	270.1 ± 20.5	267.2 ± 17.2	269.9 ± 14.6	268.4 ± 16.1	*P* = 0.948, *P* = 0.339, *P* = 0.768
	(254.3–285.8)	(254.0–280.5)	(257.8–282.1)	(255.0–281.9)	
**GCC**					
*Average (μm)*	102.8 ± 6.9	101.5 ± 7.0	97.8 ± 5.8	97.2 ± 5.0	*P* = 0.139, *P* = 0.250, *P* = 0.684
	(97.5–108.1)	(96.1–106.8)	(93.0–102.7)	(93.0–101.4)	
*Superior (μm)*	101.1 ± 6.4	101.0 ± 7.8	96.4 ± 4.9	96.5 ± 6.1	*P* = 0.149, *P* = 0.995, *P* = 0.924
	(96.1–106.0)	(94.9–107.0)	(92.3–100.5)	(91.4–101.6)	
*Inferior (μm)*	104.1 ± 7.4	101.9 ± 6.8	99.3 ± 7.3	97.9 ± 4.1	*P* = 0.163, *P* = 0.104, *P* = 0.684
	(98.4–109.9)	(96.7–107.1)	(93.2–105.3)	(94.5–101.3)	
**RNFLT**					
*Average (μm)*	103.3 ± 5.3	103.0 ± 8.7	99.4 ± 6.7	101.4 ± 9.8	*P* = 0.488, *P* = 0.608, *P* = 0.470
	(98.9–107.8)	(95.7–110.2)	(92.3–106.4)	(91.2–111.7)	
*Temporal (μm)*	73.6 ± 9.1	75.3 ± 8.3^*^	72.2 ± 9.3	75.7 ± 9.1^††^	*P* = 0.922, *P* < 0.001, *P* = 0.119
	(66.0–81.2)	(68.4–82.3)	(62.5–82.0)	(66.1–85.3)	
*Superior (μm)*	126.3 ± 6.6	122.8 ± 6.9	123.7 ± 12.5	121.7 ± 14.1	*P* = 0.716, *P* = 0.189, *P* = 0.726
	(120.8–131.8)	(117.1–128.6)	(110.6–136.8)	(106.9–136.4)	
*Nasal (μm)*	89.9 ± 18.0	87.2 ± 11.7	85.7 ± 14.3	80.6 ± 17.0	*P* = 0.482, *P* = 0.316, *P* = 0.750
	(74.9–104.8)	(77.3–97.0)	(70.7–100.7)	(62.7–98.4)	
*Inferior (μm)*	124.5 ± 12.6	124.9 ± 17.3	122.3 ± 14.5	128.4 ± 9.1	*P* = 0.917, *P* = 0.491, *P* = 0.541
	(114.0–135.0)	(110.4–139.4)	(107.1–137.5)	(118.8–138.0)	

*AMT, average macular thickness; BDC-2, 2 days before DI; GCC, ganglion cell complex; RNFLT, retinal nerve fiber layer thickness; R+1, first day after DI. Values in parentheses represent 95% CI of the mean*.

* *P < 0.05 vs. BDC-2 in control group*,

†† *P < 0.01 vs. BDC-2 in cuffs group*.

#### Average Macular Thickness by Retinal Map

Average macular thickness was not significantly modified after DI in both groups (Table 3).

#### GCC Thickness

Average, superior, and inferior GCC thickness were preserved after DI in both groups (Table 3).

#### Retinal Nerve Fiber Layer Thickness

We observed the thickest RNFLT in the temporal quadrant both in the control and cuffs groups after DI with *P* = 0.050 and *P* = 0.002, respectively; however, the average RNFLT was preserved. Neither did we notice any significant changes in RNFLT in the superior, nasal, or inferior quadrants (Table 3).

### Optic Nerve Sheath Diameter

**Figure 6:**
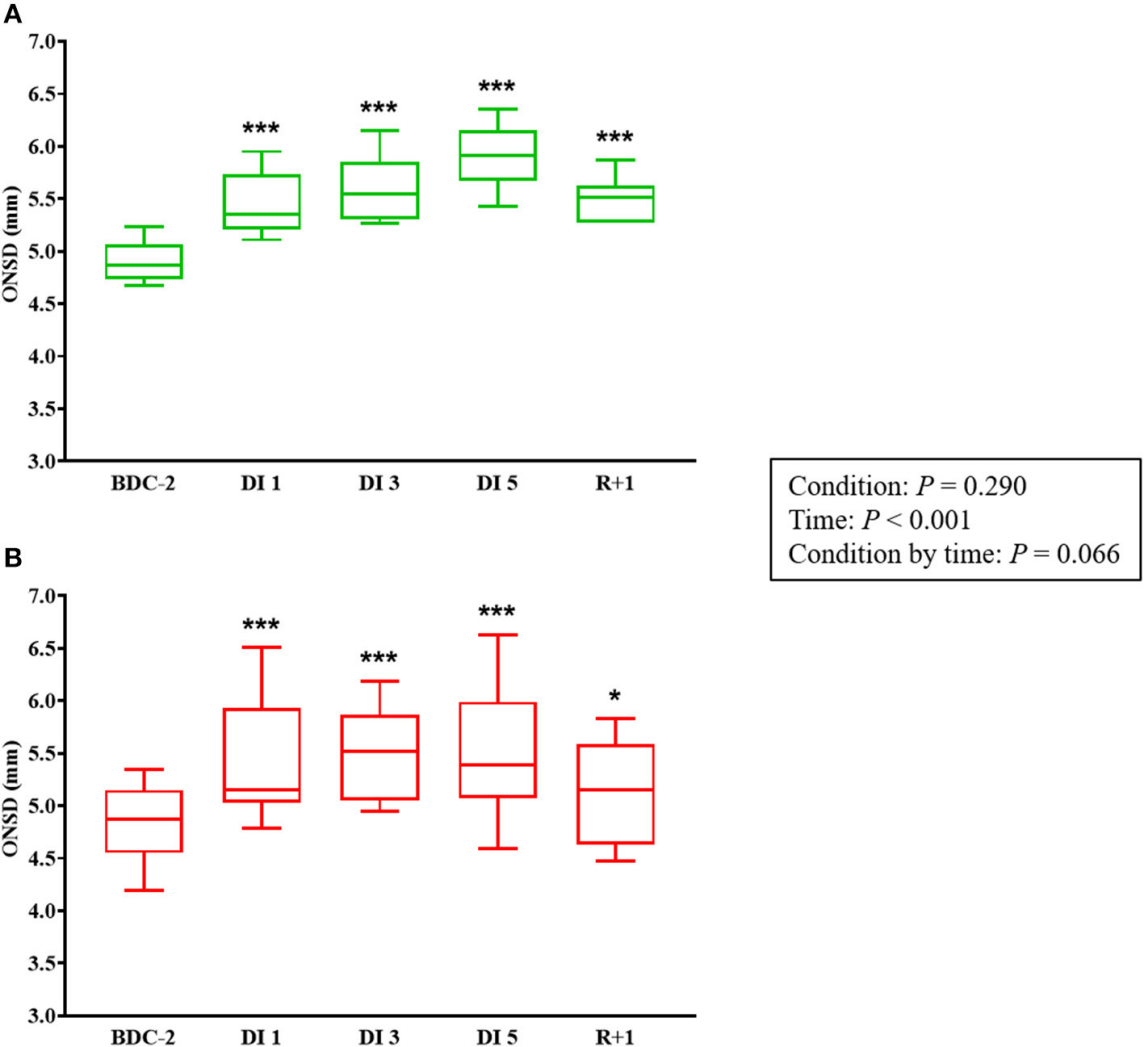
ONSD measurements before, during, and after DI in control **(A)** and cuffs **(B)** groups. BDC-2, 2 days before DI; DI 1, first day of DI; DI 3, third day of DI; DI 5, fifth day of DI; ONSD, optic nerve sheath diameter; R+1, first day after DI. *P < 0.05 vs. BDC-2, ***P < 0.001 vs. BDC-2.

Globally, VTC tended to dampen the ONSD enlargement induced by DI (*P* = 0.066). In the control group, ONSD increased significantly by 11% during DI 1 (5.4 ± 0.3 mm; *P* < 0.001), 15% during DI 3 (5.6 ± 0.3 mm; *P* < 0.001), 20% during DI 5 (5.9 ± 0.3 mm; *P* < 0.001), and 12% at R+1 (5.5 ± 0.2 mm; *P* < 0.001) vs. BDC-2 (4.9 ± 0.2 mm) (Figure 6A). In the cuffs group, ONSD increased significantly by 12% during DI 1 (5.4 ± 0.6 mm; *P* < 0.001), 14% during DI 3 (5.5 ± 0.5 mm; *P* < 0.001), 14% during DI 5 (5.5 ± 0.6 mm; *P* < 0.001), and 6% at R+1 (5.1 ± 0.5 mm; *P* = 0.024) vs. BDC-2 (4.8 ± 0.4 mm) (Figure 6B).

### Intraocular Pressure

One subject in the control group and one subject in the cuffs group were excluded from IOP data analysis due to technical glitches.

**Figure 7:**
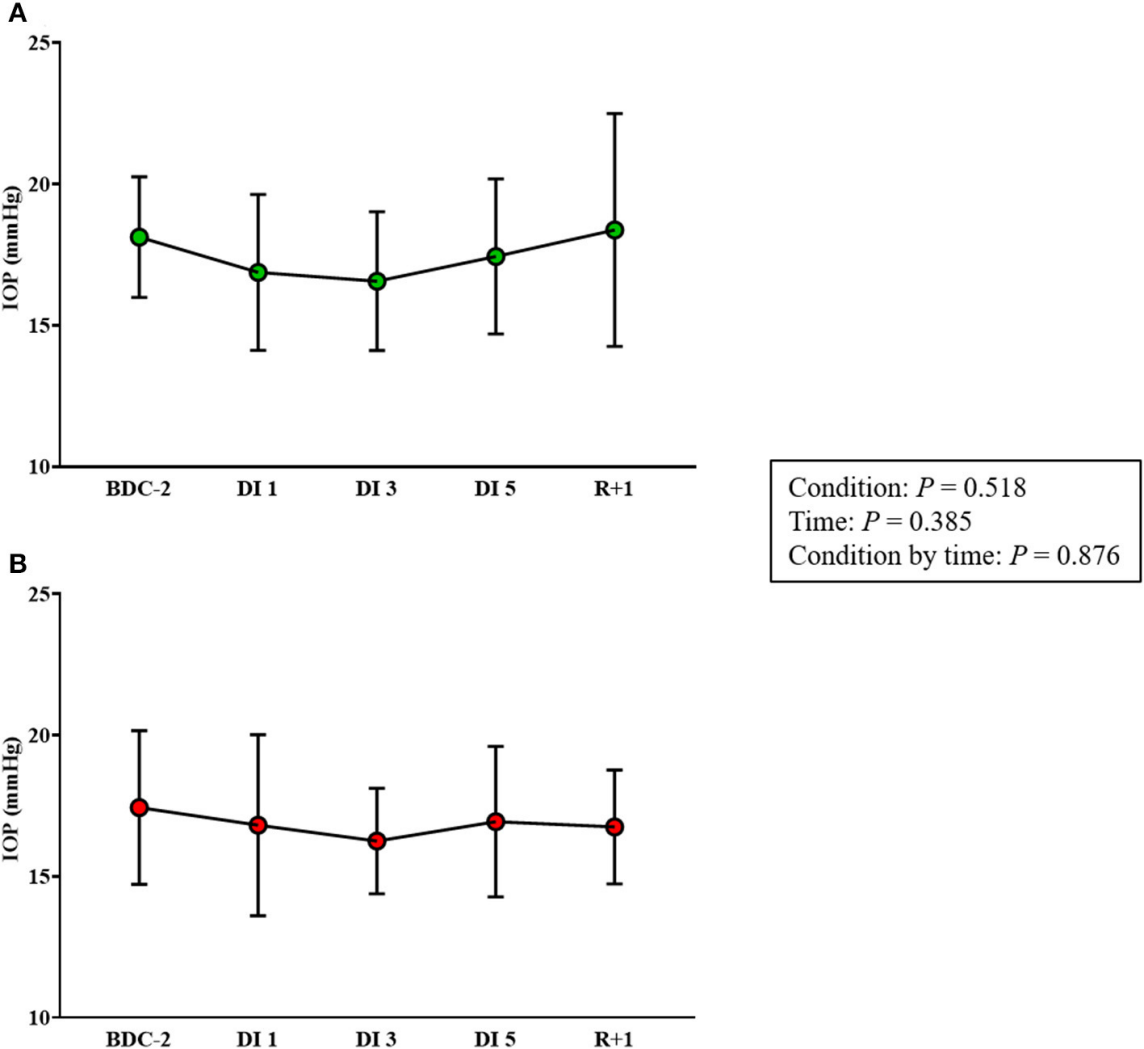
Intraocular pressure (IOP) measurements before, during, and after DI in control **(A)** and cuffs **(B)** groups. BDC-2, 2 days before DI; DI 1, first day of DI; DI 3, third day of DI; DI 5, fifth day of DI; IOP, intraocular pressure; R+1, first day after DI.

No difference was observed between the control and cuffs groups (*P* = 0.876). In the control group, IOP was not significantly modified during DI 1 (16.9 ± 2.8 mmHg; *P* = 0.392), DI 3 (16.6 ± 2.5 mmHg; *P* = 0.052), DI 5 (17.4 ± 2.7 mmHg; *P* = 0.941), and R+1 (18.4 ± 4.1 mmHg; *P* > 0.999) vs. BDC-2 (18.1 ± 2.1 mmHg) (Figure 7A). In the cuffs group, IOP was not significantly changed during DI 1 (16.8 ± 3.2 mmHg; *P* = 0.822), DI 3 (16.3 ± 1.9 mmHg; *P* = 0.654), DI 5 (16.9 ± 2.7 mmHg; *P* = 0.986), and R+1 (16.8 ± 2.0 mmHg; *P* = 0.840) vs. BDC-2 (17.4 ± 2.7 mmHg) (Figure 7B).

## Discussion

This study shows that 5 days of DI induces ophthalmological changes, such as a slight but significant increase in RNFLT in the temporal quadrant and an enlargement in ONSD. VTC countermeasures seem to have minor impacts on these ophthalmological modifications.

### Thickening of Retinal Nerve Fiber Layer in the Temporal Quadrant After DI, No Effect of VTC

Spectral-domain OCT is a fast, accurate, and non-invasive imaging technique that uses infrared light waves to take high-resolution cross-section pictures of the retina, choroid, and ONH. This method allows for the early detection of optic nerve edema. New high-resolution spectral-domain OCT technology has had a significant impact on quantifying early morphological changes of the posterior ocular structures even before the onset of symptoms or clinical signs, such as papilledema. As such, OCT has become the primary diagnostic tool for the early detection and monitoring of SANS before, during, and after spaceflight. These results indicate that 5 days of DI induces the thickest RNFL in the temporal quadrant; however, the resolution limit of spectral-domain OCT is between 5 and 10 μm. This limit could compromise the significance of this study results.

Previous studies demonstrated that astronauts presented degradation in visual acuity, such as hyperopic shift or residual choroidal folds during either short- or long-duration spaceflight (Mader et al., 2011; Lee et al., 2016). Moreover, a case report performed on a 57 year-old astronaut, who underwent 2 long-duration spaceflights, showed that the second mission has worsened the ophthalmological outcomes observed during the first mission (unilateral choroidal folds and a single cotton wool spot) (Mader et al., 2013). Lee et al. (2016) showed that 29 and 60% of crewmembers who underwent short- and long-duration spaceflights, respectively, presented degradation in distant and near visual acuity. Another case report on a healthy 45 year-old male astronaut, who spent ~6 months on the International Space Station, depicted an increase in total retinal thickness and remained high even after 1 year (Mader et al., 2017). Furthermore, in a retrospective study, Patel et al. (2018) also described ophthalmological changes in astronauts measured by OCT scans. Indeed, 15 astronauts exhibited an increase in total retinal thickness and a greater global circumpapillary RNFLT (about 20 μm) with a larger increase in the inferior RNFL quadrant after long-duration spaceflight. Macias et al. (2020) found similar findings. Indeed, 11 astronauts, who spent an average duration of 170 days on the International Space Station, exhibited an increase in global total retinal thickness that persisted throughout the mission. Macias et al. (2021) evaluated ophthalmologic changes, before, during, and after spaceflight (up to 1 year) in 11 crewmembers. Only 2 of 11 crewmembers developed ocular outcomes with an increase in total retinal thickness. The authors denoted the importance of taking into account the high interindividual variability of ocular changes encountered during long-duration spaceflight. It is noteworthy that crewmembers may develop choroidal folds and optic disc edema over 1 year.

In a 30 day HDBR study, spectralis OCT unveiled an average increase in peripapillary retinal thickness of about 19.4 μm in a healthy 25 year old male astronaut. However, no clear evidence of optic disc edema was detected (Taibbi et al., 2013). Cases of papilledema and a significant increase in peripapillary total retinal thickness have recently been described after 30 days of strict HDBR (Laurie et al., 2019). Interestingly, they also found major differences with a greater peripapillary total retinal thickness in individuals who underwent 30 day HDBR vs. astronauts during short-duration spaceflight (22 to 47 days) (Laurie et al., 2020); however, we did not find such changes in OCT data compared with HDBR and spaceflight. One assumption explaining these differences would come from the time duration of the experiment. Another hypothesis put forward by Laurie et al. (2020) is that the position of the torso would mitigate cephalad venous congestion and thus reducing ICP. Indeed, as a reminder in this study, the subjects immersed are in a semi-recumbent position. Taibbi et al. (2016) have compared the effects of a 14 and 70 day HDBR by OCT; they found that 70 day HDBR induced greater peripapillary retinal thickening than 14 day HDBR, suggesting that time might also affect the amount of optic disc swelling. In this study, these structural ophthalmological changes could be due to the elevation in ICP from microgravity-induced thoraco-cephalic fluid shift during DI.

VTC are mechanical countermeasures commonly used by cosmonauts to offset thoraco-cephalic fluid shift with reduction of facial edema and congestion (Lindgren et al., 1998). VTC have already been tested during short-term bed rest studies. Although their daily use proved their efficiency to limit hypovolemia and/or baroreflex impairment, VTC failed to prevent orthostatic intolerance (Arbeille et al., 1999; Custaud et al., 2000; Millet et al., 2000; Pavy-Le Traon et al., 2001; Robin et al., 2020); however, little is known about the impact of mechanical countermeasures on ocular changes. This study shows that VTC failed to prevent the increase in RNFLT in the temporal quadrant induced by DI.

### ONSD Enlargement During and After DI, VTC Dampened the ONSD Changes

To date, very few studies assessed ONSD in astronauts after spaceflight. For instance, Mader et al. (2011) revealed globe flattening, determined by magnetic resonance imaging (MRI) in some astronauts. Interestingly in this cohort, a persistent rise in ICP (up to 28.5 cm H_2_O corresponding to ~20 mmHg) was observed several weeks after spaceflight. Furthermore, Kramer et al. (2012) showed that after long-duration spaceflight, ONSD varied greatly according to the presence of globe flattening. Indeed, 20 astronauts with no globe flattening had a lower average ONSD (5.8 ± 0.6 mm) as opposed to a higher average ONSD (7.2 ± 1.5 mm) in 7 astronauts who exhibited globe flattening. Similar observations were reported in astronauts with nerve kinking who exhibited a greater ONSD, in contrast to those who did not, with value of 7.5 ± 1.1 mm vs. 5.9 ± 0.8 mm, respectively. Mader et al. (2013) also described that in a 57 year-old astronaut who underwent several consecutive long-duration missions to the International Space Station, an enlargement in ONSD during a post-mission examination was observed. In a study performed with 2D ultrasound, Sirek et al. (2014) measured ONSD in 13 astronauts from different cohorts, before, during, and after long-duration spaceflight. In-flight ONSD values were increased about 11% relative to pre-flight ONSD values without an immediate recovery post-flight. Mader et al. (2017) also found in a 45 year-old astronaut an increased ONSD bilaterally, measured by orbital 3T MRI, after a 6 month mission to the International Space Station. In contrast, 10 astronauts experiencing a 6 month spaceflight did not exhibit any changes in ONSD determined by quantitative MRI, suggesting that ICP did not reach a pathological threshold (Rohr et al., 2020).

It is now well-recognized that ONSD is a surrogate marker for elevated ICP (Geeraerts et al., 2007). These findings are consistent with the previous study, where we found an elevation in ONSD (up to 30%) in 12 healthy male subjects during 3 days of DI (Kermorgant et al., 2017). Ophthalmological changes are rapidly observed in DI whereas they were reported much later in HDBR. Indeed, the authors found a significant increase in ONSD at day 57 of HDBR (unpublished data in collaboration with Pr. Jean-Claude Quintyn). The normal ICP range was determined from 5 to 15 mmHg in a horizontal position (Rangel-Castilla et al., 2008). Even if no predetermined ONSD cut-off value is formally established to define intracranial hypertension (elevated ICP is defined as ICP ≥ 20 mmHg), the threshold of ONSD distension to define elevated ICP could vary between 5 and 5.9 mm (Moretti and Pizzi, 2011). Moreover, Geeraerts et al. (2008) demonstrated that ONSD values above 5.82 mm could reflect intracranial hypertension with a 90% probability. Analogously, Soldatos et al. (2008) found a cut-off ONSD value of about 5.70 mm with sensitivity = 74% and specificity = 100% for predicting high ICP. The ONSD values found in the control group could correspond to values reflecting intracranial hypertension (Geeraerts et al., 2008; Soldatos et al., 2008).

Several mechanisms may explain the enlargement in ONSD. With the presence of an increased ICP induced by microgravity-associated thoraco-cephalic fluid shift, the hydrostatic transmittance of cerebrospinal fluid traveling within the subarachnoid space may expand the retrobulbar part; thus leading to a local ONSD enlargement (Stenger et al., 2017). This enlargement phenomenon would occur before the onset of papilledema (Hansen et al., 2011). Venous-drainage pathways may play a key role in elevated ICP (Beggs, 2013). Venous-drainage impairment has been confirmed in a recent work, where 6 of 11 healthy crewmembers, who spent a mean of 210 days in spaceflight, presented stagnant or reverse flow in the internal jugular vein measured by Doppler ultrasonography (Marshall-Goebel et al., 2019). In addition, Arbeille et al. (2017; 2020) described in healthy subjects who underwent several days of DI, changes in venous redistribution, especially an increase in jugular vein volume during the first 2–3 h of DI; however, only a residual effect in jugular vein volume was found during the 4th day of DI (Arbeille et al., 2020). Still, the same authors assessed indirectly the ICP by using the cochlear response to auditory stimulation and only half of them depicted a rise in ICP suggesting that an increased venous pooling was not the only criterion explaining an increased ICP (Arbeille et al., 2017). What pleads in favor of this assumption is the fact that cerebrospinal fluid may have been compartmentalized and sequestered in the orbital subarachnoid space. Impaired venous and lymphatic drainage may alter cerebrospinal fluid absorption within the orbit leading to a distended subarachnoid space (Geeraerts et al., 2008; Stenger et al., 2017). Subjects genetically predisposed may also develop ophthalmic outcomes. For instance, Zwart et al. (2012) demonstrated differences in circulating concentrations of the folate and B12-dependent 1-carbon metabolic pathway between crew members.

While we did not observe significant differences in ONSD between control and cuffs groups, VTC seem to alleviate microgravity-induced ONSD changes. Indeed, ONSD values in the cuffs group may indicate a moderate rise in ICP (Geeraerts et al., 2008; Soldatos et al., 2008). As we previously hypothesized, the venous fluid shift may be involved in ONSD enlargement. Thus, VTC should sequester venous flow in the lower limbs and may limit venous congestion. Furthermore, it has been demonstrated that during short-duration spaceflights, thigh cuffs decreased cervico-cephalic hemodynamics with a reduction of venous stasis (Fomina et al., 2004).

### No Effect of DI on IOP

An 8 day German Spacelab mission revealed an increase in IOP about 5 mmHg immediately after exposure to weightlessness (Draeger et al., 1993). Similar results were found in a 10 day Spacelab D2 mission. Indeed, IOP was increased by ~22–23 mmHg in the early phase of the launch, remained high for 1 day but returned to pre-flight values on the 4th day (Draeger et al., 1994). A compilation of IOP data from 6 shuttle missions and performed on 11 astronauts showed similar trends (Stenger et al., 2017). Although an increase in IOP after entering microgravity was observed, these findings suggested a normalization of IOP values either after short- or long-duration spaceflight despite a maintained cranial venous fluid shift (Huang et al., 2019).

In this study, we found a decrease in IOP during DI, yet, this change did not reach significance and remained unchanged after DI. Consistently, Chiquet et al. (2003) reported in a 7 day HDBR, which was performed in young healthy volunteers, a drop in IOP associated with hypovolemia related to cephalad fluid shifts produced by HDBR. The authors suggested that these ocular changes were mainly due to ocular dehydration or to systemic cardiovascular and hormonal variations during HDBR. The measurement of IOP in the same study is important since the pressure gradient between ICP (which would increase) and IOP (which would remain stable or decrease) may be one of the factors favoring optic nerve edema (Jóhannesson et al., 2018). A case report performed on a 25 year-old Caucasian man who underwent a 30 day HDBR displayed the same trend with a diminution in IOP. This phenomenon could contribute to a decreased translaminar pressure (Taibbi et al., 2013). In contrast, Taibbi et al. showed that 14 and 70 day HDBR provoked an increase in IOP, respectively, of +1.42 and +1.79 mmHg but returned to baseline values after HDBR. The magnitude of the increase observed during HDBR was not associated with the campaign durations (Taibbi et al., 2016).

Subjects wearing VTC did not exhibit any modifications in IOP. In a recent report, acute use of VTC during a 15° tilt reversed tilt-induced increased IOP and subfoveal choroidal thickness (Balasubramanian et al., 2018). In this study, VTC is likely to tend to limit the reduction in IOP by preventing hypovolemia (previously demonstrated in this same study by Robin et al., 2020) thus diminishing ocular dehydration.

## Conclusion

Overall, DI provoked ophthalmological changes, such as the thickest RNFL in the temporal quadrant and enlargement in ONSD. These changes seem to occur more rapidly than during HDBR. Primarily, VTC had few impacts on the DI-induced neuro-ophthalmological changes. Further studies will require the identification of the underlying mechanisms and the kinetics involved to fully understand the physiological responses of DI on the ophthalmological changes. The development of an optimized countermeasure needs to be further studied to assess and mitigate the ocular changes induced by microgravity. Finally, DI can be considered as an efficient model simulating neuro-ophthalmological changes observed after short-term exposure to microgravity; however, it is difficult to extrapolate these findings on the neuro-ophthalmological consequences of long-term exposure to microgravity.

### Limitations

Limitations must be acknowledged in this study since that could affect these findings: (1) The sample size in this study could dampen the statistical significance of these results; however, the subjects served as their own control. (2) The low resolution of the OCT device could affect these findings. (3) The standard OCT examination in clinical uses or different studies is usually performed in the upright sitting position. The effects of position on the measurement, especially, on quadrant segmentation are unknown. The change of position is likely to alter RNFLT values; however, the measurements have been conducted in a semi-recumbent position, similar to that found during DI. (4) Subjects wore VTC for only 10 h per day; however, a longer application of VTC may induce detrimental venous effects. (5) The out-of-bath time performed for reasons related to the health of the subject may affect these findings by reducing the headward fluid shift and thus limiting ophthalmological changes.

## Data Availability Statement

The raw data supporting the conclusions of this article will be made available by the authors, without undue reservation.

## Ethics Statement

The studies involving human participants were reviewed and approved by both the CPP Est III Ethic Committee (October 2, 2018) and the French Health Authority, ANSM (August 13, 2018). The patients/participants provided their written informed consent to participate in this study.

## Author Contributions

AP-LT and VS equally supervised the research. M-PB, AB, BG, AG, AP-LT, and VS designed the research. MK, AS, JL, F-PR, NB, AB, BG, AG, and AP-LT performed the research. MK, AS, TG, FV, NN, J-MS, AP-LT, and VS analyzed the data. MK, AS, TG, FV, AG, NN, DA, OH, J-MS, AP-LT, and VS wrote and revised the manuscript. All authors contributed to the article and approved the submitted version.

## Conflict of Interest

The authors declare that the research was conducted in the absence of any commercial or financial relationships that could be construed as a potential conflict of interest.
